# Face Feature Change Detection Ability in Developmental Prosopagnosia and Super-Recognisers

**DOI:** 10.3390/brainsci14060561

**Published:** 2024-05-30

**Authors:** Jodie Davies-Thompson, Daniel Morgan, Josh P Davis, John R. Towler

**Affiliations:** 1 School of Psychology, Vivian Tower, Faculty of Medicine, Human & Health Sciences, Swansea University, Swansea SA2 8PP, UKj.r.towler@swansea.ac.uk (J.R.T.); 2 University of Greenwich, London SE10 9LS, UK; j.p.davis@greenwich.ac.uk

**Keywords:** faces, prosopagnosia, super-recognisers

## Abstract

In non-clinical populations, facial features (eyes, nose, mouth) may vary in their contribution to face identity perception. Changes to whole faces are easier to detect than changes to individual features, and eye changes are typically easier to detect than mouth changes, which in turn are easier to detect than nose changes. However, how this differs for people with face recognition difficulties (developmental prosopagnosia; DP) and for individuals with superior face recognition abilities (super-recognisers; SR) is not clear; although findings from previous studies have suggested differences, the nature of this difference is not understood. The aim of this study was to examine whether differences in the ability to detect feature changes in DPs and SRs were (a) quantitative, meaning that the pattern across feature changes remained the same but there was an overall upwards or downwards shift in performance, or (b) qualitative, meaning that the pattern across feature changes was different. Using a change detection task in which individual face features (eyes, nose, mouth) changed between sequentially presented faces, we found that while prosopagnosics showed a quantitative difference in performance with a downwards shift across all conditions, super-recognisers only showed qualitative differences: they were better able to detect when the face was the same and were marginally (but not non-significantly) worse at detecting when the eyes changed. Further, the only condition which distinguished between the three groups was the ability to identify when the same face was presented, with SRs being better than controls, and controls being better than DPs. Our findings suggest that, in feature-matching tasks, differences for DPs are due to them being overall worse at the task, while SRs use a qualitatively different strategy.

## 1. Introduction

There are very large individual differences in human face identity recognition ability (e.g., [1,2]). Developmental prosopagnosics (DPs) inhabit the extreme low end of the ability spectrum, while so-called super-recognisers (SRs) occupy the top. Faces comprise different internal features (e.g., eyes, nose, mouth) which vary in their prominence. Indeed, previous research has shown that there is a “feature-salience hierarchy” in terms of the ease with which facial features are recognised and manipulations to those features are detected. Using unfamiliar face-matching paradigms, participants most easily detect changes that are made to the eyes, followed in turn by changes to the mouth and then the nose [3,4,5,6,7,8,9]. Recently, Lai et al. (2014) demonstrated similar “feature-saliency hierarchy” effects using a sequential face feature change detection procedure [10]. In the present study we aim to recruit super-recognisers, controls with ability in the typical range, and developmental prosopagnosics, to determine whether individual differences in face recognition ability might be associated with qualitative variations in the face feature-salience hierarchy, or instead by a general reduction or improvement in performance irrespective of the feature being tested.

Prosopagnosia is a condition which presents as marked impairments in the ability to recognise faces even if familiar (i.e., close acquaintances). While acquired prosopagnosia is typically caused by brain trauma, lesions, or strokes affecting occipitotemporal or anterior temporal regions (see [11,12] for reviews), developmental prosopagnosia (DP) is a lifelong neurodevelopmental impairment which occurs in the absence of brain damage or other cognitive impairments [13]. Further, while acquired prosopagnosia is extremely rare, DP is estimated to affect approximately 2–3% of the population [14,15]. Studies have found that some DPs are selectively impaired in face identity as compared to facial expression recognition [16,17] and voice recognition [18], which is line with a vast range of studies showing distinct, but overlapping, mechanisms and brain areas involved in facial recognition, emotion recognition, and voice recognition (i.e., [19,20]); however, other studies have found positive correlations between unfamiliar face and voice recognition abilities in both DPs and SRs [21]. Many studies have investigated face perception in developmental prosopagnosia, yet few have measured the ability to recognise or detect changes to individual facial features.

One strategy that measures the ability to match separate face parts is the part–whole task, in which participants must match faces in which either the nose, mouth, or eyes change in isolation or in the context of a whole face. Using the procedure, in contrast to controls, DPs do not show an advantage in presenting feature changes in the context of a whole face [22], suggesting an impairment in a holistic style of face perception which makes use of the context of a whole face in a single glance (e.g., [8,23]). A further study using the part-whole task found DPs had an overall drop in performance across features, but the pattern for detecting eye changes > mouth changes > nose changes was the same for DPs and controls [24]. Taken together, these results suggest that, while differences exist between DPs and controls for holistic face processing, the feature-saliency pattern may remain the same, with an equal drop in performance across all conditions for DPs. Alternatively, other studies have suggested that DPs have an impairment in processing the eyes above and beyond other facial features; for example, an ERP study showed that contrast changes were particularly impaired when they occurred in the eye region compared to other parts of the face [25]. Furthermore, an eye-tracking study showed that DPs paid less attention to the eyes than control participants [15]. The current study aims to test these two competing hypotheses for face perception impairments in DP.

While DPs have impaired face-recognition abilities compared to the average population, others have face recognition abilities that are far above the average. These ‘super-recognisers’ (SRs [26]) are able to identify and perceive familiar and unfamiliar faces across large variations in lighting, viewpoint, expressions, and age-changes with an unusually high degree of accuracy (e.g., [26,27,28,29]). Few studies have examined feature processing ability for separate features in super-recognisers (SRs). Using the part–whole task, Belanova and colleagues found that SRs were significantly better at detecting feature changes than controls, with a subgroup of SRs performing better on the nose condition [30]. However, it is important to note that some SRs showed significant effects in the opposite direction, suggesting the underlying mechanisms supporting this skill may substantially differ, which may explain the variation in outcomes both within and between studies. This supports the finding by an eye-tracking study that showed half of SRs fixate more on the nose area than controls [31]. The SR nose dominance effect may, however, be an optimal fixation point in the centre of the image from which to more easily take in the other facial features in a single glance. Indeed, a more recent study by Dunn and colleagues suggested that, unlike typical-ability controls who tend to fixate more on the eyes, SRs employ a flexible eye gaze mechanism which the authors likened to an adjustable spotlight [32]. This mechanism appears to automatically fixate on the most salient or idiosyncratic aspect of an individual face that will support subsequent accurate identity recognition. As such, dependent on the face under observation, fixation could be on individual facial features, component parts of features, or even on the whole face. In studies involving multiple face displays, it is likely that the combined fixation data regress to the central regions of the face, explaining the nose advantage results of Bobak et al. (2017) [31]. Overall, these findings suggest SRs may have either a general increase in face feature change detection ability, or alternatively they might have a qualitative difference in the way that they view faces in terms of being more likely to fixate in the centre of the image or on the nose, as compared to control participants who tend to fixate closer to the eyes or the nasion. However, SRs show greater variation in the direction of eyes for each face, which may explain idiosyncratic response patterns across SRs.

A recent study examining familiar faces found that, similar to controls, both SRs and DPs were more sensitive to changes in “critical” features (i.e., eyebrow thickness, eye shape, eye colour, lip thickness) than changes to “non-critical” features (face proportion, eye distance, mouth size, skin colour) [32], adding weight to the idea that there are qualitative similarities in the way that these individuals perceive faces. However, it is unclear how the responses to the individual “critical” feature changes (i.e., eye changes versus nose changes) differed between DPs and SRs. We expand upon this here by directly comparing face feature change detection performance across DPs, typical-ability controls, and SRs for changes in individual features. The aim of this study is therefore to use a feature change detection task to examine the ability of members of these groups to identify changes in the whole face, the eyes, the nose, the mouth, or whether they are viewing the same face image [10]. More specifically, we examine whether, compared to controls, DPs and SRs display either quantitative differences in face feature change detection ability across the spectrum of face-recognition ability, or whether there are more qualitative differences concerning the importance of the eye region for face recognition or the utility of fixating on the nose to take in more of the face image. In order to achieve these goals, we will collect accuracy and response-time data (and a combined balanced integration measure) across three age-matched groups of SRs, DPs, and controls likely to possess typical-range levels of face-recognition ability.

## 2. Methods

### 2.1. Participants

The current study included three age-matched participant groups consisting of 33 developmental prosopagnosics (DPs), 33 super-recognisers (SRs), and 33 controls. The DPs were recruited from the Swansea University’s Prosopagnosia Database, the SRs from the University of Greenwich Face and Voice Recognition Lab’s volunteer pool, and the controls via social media. Written informed consent was obtained for all participants and the study was approved by Swansea Universities Human and Health Science ethical protocols (reference: 2022-2769-4661). Participants were not paid to take part in this study. Table 1 shows the participant demographics who took part in the study.

All participants had intact vision, no other reported cognitive difficulties, and no history of neurological trauma. Participants eligible for the DP group reported impaired face recognition in daily life, scores two standard deviations (SD) below the population mean on the PI20 (30), and pre-study impaired performance on at least two out of three standardised tests of face recognition. These tests were the CFMT [15], the CFPT [33,34], and a modified version of the Famous Face Test (FFT [31]).

Published SR group allocation had been scored approximately 2 SDs above the mean score from representative samples (i.e., [29,35]) on both the Cambridge Face Memory Test: Extended (CFMT+) [26] and the GFMT [35], achievable by less than 2.5% of the population (e.g., [20,36]). As the GFMT has low sensitivity, a more robust standard was applied, in that minimum inclusion criteria for SRs were scores approximately two SDs above the mean on the CFMT+ and at least one other test from a choice of three: the GFMT, the Glasgow Face Matching Test: Version 2 (GFMT2HI) [37], and the Adult Face Memory Test (AFMT) [38]. Furthermore, SRs also needed to achieve scores 1.5 SDs above the mean on the Kent Face Matching Test (KFMT) [39,40,41], a sensitive face recognition ability measure for the top of the spectrum. Out of the 33 SRs, 5 achieved the minimum standard only. The other 28 SRs exceeded standards. Indeed, four, ten, and eleven achieved a conservative 2 SDs above the mean thresholds on five, four, and three tests respectively, while a further four SRs achieved conservative standards on two tests, and liberal standards on another two tests.

Control participants were recruited from the general population and via social media, reported no day-to-day difficulties in face recognition, and scored within the normal range (within 2 SDs from the mean) on the PI20, CFPT, CFMT, and FFT.

### 2.2. Independent Face-Recognition Tests

Face-processing abilities were measured using the Cambridge Face Memory Test (CFMT), the Cambridge Face Perception Test (CFPT), and the Famous Face Test (FFT). The CFMT [13] measures memory of newly learned faces. It is widely used in the study of individual differences in face recognition [42]. Participants are required to memorise a series of six different male faces and subsequently identify the learned face among three faces in each trial (72 in total). The CFMT increases in difficulty across trials, with modifications of lighting and the viewpoint from which the face is seen, as well as the addition of visual noise.

The CFPT [43] measures the ability to distinguish small differences between faces. In 16 trials, participants are asked to sort a series of six faces according to their resemblance to a target face. Stimuli are versions of the target face morphed with other faces at six different levels (28–88%). Faces are inverted in half of the trials. The final score corresponds to the distance between the sequences produced by participants and the correct sequences and is calculated separately for trials with upright and inverted faces. A larger score indicates a greater distance and, hence, poorer face-perception abilities. Finally, to test participants’ abilities to recognise familiar faces, modified versions (for British participants) of the Famous Face Test were used.

Two different versions of the FFT were used—one for 18–34-year-old participants, and another for 35+ year olds [31]. Each version presents 60 famous faces in a random order and for unlimited time. Participants were asked to provide the name of the celebrity, or biographical information if participants could not name them. If participants could not identify a face, they were then told the celebrity’s name and asked if they had previous exposure to that person. Celebrities who were not known to the participant by name were removed from the scoring for that participant.

Five tests were available to test super-recognisers: the Cambridge Face Memory Test: Extended (CFMT+) [26], the Glasgow Face Matching Test [35], Glasgow Face Matching Test 2-High [44], the Kent Face Matching Test [39], and the Adult Face Memory Test (AFMT).

To ensure that the 102 trial CFMT+ was suitable for super-recognisers, the authors added 30 extra trials at the end of the original CFMT [13]. In this final block, white noise was added, facial expressions varied more substantially, and distractors were repeated more often in order to increase task difficulty.

The GFMT [35] is a 40-trial, equally numbered simultaneous face comparison test in which participants view two facial images on a screen and decide whether they depict the same person or two different people. There are equal numbers of same-face and different-face trials. All images of faces were taken on the same day and are the same size on the screen.

The GFMT-High Version 2 [37] is an updated 40-trial version of the GFMT [35] using alternative images from the same database as the original. The authors created three different versions of the test. For this research, the “High” difficulty version was employed as it successfully addresses the ceiling effects and lack of sensitivity found when the original GFMT is taken by SRs (e.g., [45]).

The KFMT [39] also has exactly the same design as the GFMT except that the images of the same person in each pair were taken several months apart and the height of one of the two images on the screen is 1.5 times greater than the second image.

The AFMT [38] requires participants to familiarise themselves with 30 male faces, shown sequentially for 8 s each in a learning phase. In the test phase, they view 60 sequentially presented faces, 30 of which are different same-day, different-clothing images of the faces shown in the first phase. They are asked to respond “Old” or “New” to each face.

### 2.3. Experimental Stimuli

**Figure 1:**
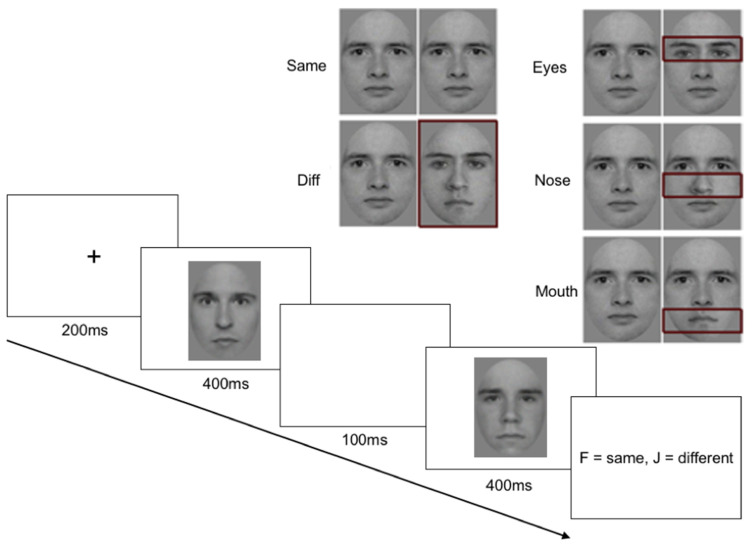
Methods. Examples of stimuli used in the different conditions. For illustrative purposes only, the red rectangles indicate the image component that was changed in each condition. In the “same” condition, two identical face images were presented, while in the “different” condition, two different identities were presented. In the feature conditions, either the eyes, nose, or mouth changed between image pairs. Participants were presented with a face pair and asked whether the two images were the “same” or “different”. There were 240 trials in total (48× each of the five conditions) presented in a pseudorandom order.

To determine the contribution of different face parts (e.g., eye, nose, and mouth region), five face-pair conditions (different face, same face, eyes different, nose different, and mouth different) were included. Stimuli were used in a previous published paper [10] and face images were obtained from the HVEM database. Participants who had their face images included in the HVEM database completed a consent form for use of their images in for research purposes and were compensated for their time. In the “same face” condition, the first and second faces were identical, while in the “different face” condition, two different faces were presented. In the “eyes different” condition, a horizontal feature band containing only the eyes differed between the two faces. In the “mouth different” condition, only a band containing the mouth differed, while in the “nose different” condition, only a band containing the nose differed. The first and the second image presented in a pair different in size by 10% (see Figure 1 for examples).

Stimuli included eight photographs of neutral, frontal-view faces of young Caucasian males. Images were grayscale, matched for luminance, and distinguishing features (such as blemishes, moles, facial hair) were removed. A grey aperture mask was placed over each face in order to remove external features (hair, chin, and ears), resulting in an oval facial image of 547 pixels in height and 400 pixels in width. For more information see [10].

### 2.4. Feature Change Detection Experiment

DPs who met criteria (i.e., scoring two SDs below the mean on the PI-20 on at least two independent neuropsychological tasks) were invited to take part in the online study via email containing a link to Gorilla Experiment Builder (www.gorilla.sc, accessed on 30 April 2024; [46]). The experiment was restricted to computers and laptops (participants could not use phones or tablets). The experiment link led to an information sheet, a demographics questionnaire, and the experimental instructions. Prior to taking part in experimental trials, participants took part in 5-pair practice trials (one of each condition) to ensure they understood the experiment. Each trial consisted of two faces presented sequentially for 400 ms each, separated by an inter-stimulus interval of 100 ms, followed by a participant response (Figure 1). To reduce contributions from low-level retinotopic processes, the second face within a pair was decreased in size by 10%. The task was to respond whether the faces were the same or different (F = “same”, J = “different”), and participants were instructed to respond as accurately and as quickly as possible (note that given the rapid stimulus presentation, participants naturally responded quickly after the stimulus duration was over). There were 240 total trials (48 trials per each of the five conditions) presented in a pseudorandom order. Reaction times and accuracy scores were collected for each trial.

### 2.5. Statistical Analysis

To determine whether face-recognition ability impacted feature change detection, we collected accuracy and response-time data on each trial from all participants. Only reaction-time data for correct trials were included in the analysis. To take into account the possibility of speed-accuracy trade-offs, accuracy and reaction time data were combined to obtain a single measure using balanced integration scores [47,48,49]. These scores weighted accuracy and reaction times equally by converting each to z-scores based on performance across all participants combined, and then subtracting the reaction time z-score from the percent correct z-score for each participant. Balanced integration scores have previously been shown to be more sensitive at identifying individuals with face-recognition problems than using accuracy alone [49]. See Liesefeld et al. (2015) for the formula used and a detailed explanation of the formula [47].

Next, balanced integration scores (BSI) for each condition and for each participant were entered into repeated-measures ANOVAs to test for main effects of Group (controls, DPs, SRs), Condition (different face, same face, different eyes, different nose, different mouth), and interactions. These were then followed up with post-hoc *t*-tests.

## 3. Results

**Figure 2:**
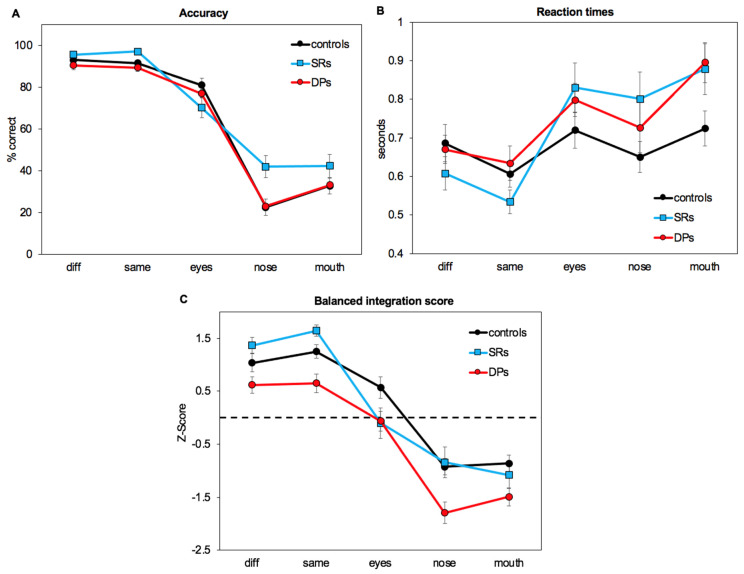
Accuracy (**A**), reaction times (**B**), and balanced integration scores (BSI) (**C**) from the three participant groups (controls, developmental prosopagnosics (DP), and super-recognisers (SR)) on each of the five conditions.

Figure 2 shows the mean accuracy, response times, and balanced integration scores (BIS) from the three participant groups (controls, DPs and SRs). Based on accuracy and response times, all groups were better for the different and the same-face changes than detecting when individual features changed. While examining only accuracy scores, DPs and controls showed similar performance across conditions, while super-recognisers performed better on the nose condition than both groups. For reaction times, all groups were slightly faster at identifying nose changes than eye and mouth changes. As planned a priori, and due to the less accurate scores for the nose condition together with the faster responses suggesting speed-accuracy trade-offs, we analyse both the accuracy scores (as typically used in previous papers, and to allow readers to compare findings across our study and other studies) and the balanced integration scores. Complete statistical analysis of reaction times, including tables, are included as Supplementary Materials, but a brief summary is included below.

### 3.1. Accuracy

A 3 (group; between subjects) × 5 (condition; within subjects) ANOVA revealed significant main effects of Group (F(2,96) = 4.65, *p* = 0.001), Condition (F(4,384) = 257.47, *p* < 0.001), and a significant interaction (F(8,384) = 3.19, *p* = 0.002). As we were interested in how the two groups (DPs and SRs) differed in their response to controls, we examined these differences by conducting 2 × 5 ANOVAs for SRs vs. controls, and another for DPs vs. controls.

### 3.2. Super-Recognisers vs. Controls

For the SRs vs. controls ANOVA, effects of Group (F(1,64) = 4.41, *p* = 0.04), Condition (F(4,256) = 149.51, *p* < 0.001) and the Interaction (F(4,256) = 5.01, *p* < 0.001) were significant. To further investigate the significant Interaction, we conducted two one-way ANOVAs on the condition variable, separately for controls and SRs. The results were significant for controls (F(4,128) = 122.00, *p* < 0.001) and SRs (F(4,128) = 46.10, *p* < 0.001), although effect sizes were stronger in controls. Tukey’s post-hoc *t*-tests (Table 2) revealed significant differences between the five conditions for SRs (P’s < 0.001), with the exception of the same vs. different comparison, and the nose vs. mouth comparison (P’s = 0.99). The analyses with controls revealed identical outcomes to SRs, except the same-face and eyes comparison was also not significant. Controls showed a very similar pattern to SRs with significant differences between all conditions (P’s < 0.05), but no differences between the same and different conditions, or between the nose and mouth (P’s > 0.13); however, controls also showed no difference between the same-face and eye changes (*p* = 0.12).

The Interaction between SRs and controls (Table 3) was caused by a greater accuracy to the same condition for SRs than controls (*t*(64) = 3.31, *p* < 0.001) as well as the nose condition (*t*(64) = 2.99, *p* < 0.001). There were no other significant differences (P’s > 0.08).

In sum, these results therefore showed that SRs were more accurate on the same-face condition as well as the nose-change condition, suggesting qualitative differences between SRs and controls.

### 3.3. Developmental Prosopagnosics Compared to Controls

For DPs versus controls, there was an effect of Condition (F(4,256) = 239.80, *p* < 0.001), but no effect of Group (F(1,64) = 0.74, *p* = 0.39) or Interaction (F(4,256) = 0.21, *p* = 0.93). The effect of Condition was explored using Tukey’s post-hoc *t*-tests collapsed across groups (Table 4) which showed significant differences between all conditions (P’s < 0.05) with the exception of no accuracy differences between the same and different conditions (*p* = 0.99). In sum, controls and DPs showed similar patterns of accuracy.

### 3.4. Response Times

Complete statistical analysis of reaction times, complete with tables, is included in the Supplementary Materials. However, we include a brief summary here. When comparing controls and SRs, there was no effect of Group, but an effect of Condition and Interaction. SRs had no significant differences in reaction times between the eyes, nose, and mouth, or between whole face changes or the same face, while controls only showed differences in reaction times between the same face and eye changes, and the same face and mouth changes. When comparing controls to DPs, there was an effect of Group and Condition, but no Interaction. Controls had overall faster reaction times than DPs. Both controls and DPs had faster reaction times for same face than for eye changes, nose changes, and mouth changes, as well as faster reaction times for different face than mouth changes.

### 3.5. Balanced Integration Analysis

Next, we examined the balanced integration scores (BSI) which take into account speed-accuracy trade-offs. A 3 (group; between subjects) × 5 (condition; within subjects) ANOVA revealed a main effect of Group (F(2,96) = 6.24, *p* = 0.003), Condition (F(4,384) = 127.40, *p* < 0.001), and an Interaction (F(8,384) = 2.26, *p* < 0.001). As we were interested in how the two groups (DPs and SRs) differed in their response to controls, we examined these differences by conducting 2 × 5 ANOVAs for super-recognisers versus controls, and another for prosopagnosics versus controls.

### 3.6. Super-Recognisers Compared to Controls

For SRs versus controls, there was no effect of Group (F(1,64) = 0.01, *p* = 0.94), but an effect of Condition (F(4,256) = 125.94, *p* < 0.001) and an Interaction (F(4,256) = 4.84, *p* < 0.001). The effect of Condition was explored using Tukey’s post-hoc *t*-tests (Table 5) which showed significant differences between all conditions for both controls and SRs (P’s < 0.05), with the exception of no differences between the same and different conditions, or between the nose and mouth (P’s > 0.68). This replicated the findings by Lai et al. (2014), which found that eye changes were easier to detect than nose and mouth changes for both groups (P’s < 0.05), but no difference between nose changes and mouth changes (P’s > 0.84).

The Interaction between SRs and controls (Table 6) was caused by a greater response to the same condition for SRs than controls (*t*(64) = 2.41, *p* = 0.02). There was also a trend for controls to be better than SRs for the eye condition (*t*(64) = 1.63, *p* = 0.06). There were no other significant differences (P’s > 0.15).

In sum, these results therefore showed no quantitative differences between SRs and controls with no overall differences in performance, but instead a qualitative difference with SRs better able to detect when the face was the same and marginally worse at detecting when the eyes changed.

### 3.7. Developmental Prosopagnosics Compared to Controls

For DPs versus controls, there was an effect of Group (F(1,64) = 12.7, *p* < 0.001) and an effect of Condition (F(4,256) = 142.76, *p* < 0.001) but no Interaction (F(4,256) = 0.79, *p* = 0.53). The effect of Group with no Interaction was caused by an overall better performance for controls than prosopagnosics across all conditions; however, we have included these comparisons for reference in Table 7.

The effect of Condition was explored using Tukey’s post-hoc *t*-tests collapsed across groups (Table 8), which showed significant differences between all conditions (P’s < 0.001), with the exception of no differences between the same and different conditions, or between the nose and mouth (P’s > 0.62).

In sum, these results confirmed the observed general reduction in performance for DPs compared to controls across all feature-change conditions. Separate analyses of accuracy and response times showed that these pervasive differences on the task were mainly accounted for by DPs’ slower response times.

### 3.8. Individual Differences

**Figure 3:**
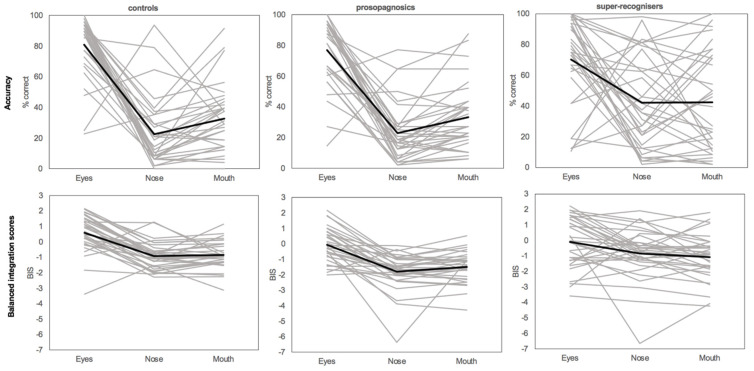
Individual differences in accuracy and balanced integration scores across the three participant groups for the feature changes. Grey lines represent individual participants while the black line represents the average for each group.

Figure 3 shows the individual results for each participant on the individual feature changes (eyes, nose, mouth) for accuracy and balanced integration scores. As can be seen in the figure, the controls and DPs showed similar patterns and were relatively consistent across participants. However, the SRs showed much larger variations in their responses—particularly for accuracy. While the average and most SRs showed better accuracy for eyes and similar performance for nose and mouth, some SRs showed greater accuracy for nose changes (this in line with previous findings; [21]). When reaction times were accounted for in the balanced integration scores, the variability became less; this suggests that SRs had significant variability and heterogeneity across their speed–accuracy trade-offs, and that individual SRs may be using different strategies to identify feature changes.

### 3.9. Results Summary

DPs showed an overall drop in performance across conditions but no differences in the pattern of responses across conditions, and these differences were mainly accounted for by DPs slower response times. On the other hand, SRs showed no overall differences in performance but were specifically better able to detect when the face was the same and marginally worse at detecting when the eyes changed. Interestingly, SRs were also more accurate at detecting nose changes than control participants, but this increase in accuracy was accompanied by slower response times in this condition (as well as for the other feature-change conditions), suggesting a more complex pattern of behaviour. The only condition which yielded significant differences both between DPs and controls, and between SRs and controls, was the ability to identify when the same face was presented, with SRs being better than controls, and controls being better than DPs.

## 4. Discussion

The aim of this study was to examine whether developmental prosopagnosics and super-recognisers showed differences in their ability to detect face feature changes compared to controls. Specifically, we asked whether any differences in the ability to detect face feature changes were quantitative, meaning that face feature detection performance would be generally better or worse across all features tested, or qualitative, meaning that specific patterns emerged that distinguished between the different groups. For example, these could include feature-specific hypotheses involving a proposed eye-processing deficit in DP, or a “nose-superiority” effect for SRs. Overall, we found that prosopagnosics showed a quantitative difference with an overall drop in performance across conditions but no differences in the patterns of responses across conditions. On the other hand, super-recognisers showed qualitative differences, with greater accuracy for detecting nose changes than controls, and were also better able to detect when the face was the same. Furthermore, the only condition which reliably distinguished between the three groups was the ability to identify when the same face was presented, with SRs being better than controls, and controls being better than DPs. These findings will be discussed in detail below.

Our findings for our control group largely replicate those reported by Lai et al. (2014), who found better performance when the whole face changed as compared to individual feature changes, and better performance for eye changes compared to nose or mouth changes [10]; however, in our participant sample, there was no difference between nose and mouth. One possible explanation for this difference is that our participants were older than the participants used in the Lai study (mean age = 42.6, compared to mean age = 22.9). Indeed, one study examining the effect of participants’ ages on face matching and face memory found that worse performance on a face-memory task could be explained by the reduced performance on the face-matching tasks [50]; further, studies have reported specific deficits in face-feature processing with increasing age (e.g., [50,51]).

We found that DPs showed an overall drop in performance across all conditions compared to controls, but no difference in the pattern of response across conditions between the two groups. In other words, DPs showed the same feature effects as controls but were just overall worse at detecting face-feature changes (and repetitions). These differences primarily stemmed from slower response times than the control group, suggesting a lower level of confidence in the DP or that a more time-consuming strategy may have been employed. Our findings fit with those from a previous study which found that DPs had an overall drop in performance for feature changes, but the pattern for detecting eye, mouth, and nose changes was the same for DPs and controls [24]. This was also found in another study examining the ability of DPs and controls to detect changes in “critical” features and “non-critical” features, where DPs showed similar patterns of response to controls [32]. One study which used eye tracking to measure scan paths during a face memory task showed no difference in scan paths of faces in DPs and controls, with both groups showing a bias towards the eyes over the mouth [52] (for different findings, see [31]). Taken together with our results, this suggests that DPs may be impaired in detecting changes to all internal facial features rather than having a specific or pronounced deficit for the eye region.

Our results showed that SRs were not significantly better than controls on our task overall, but instead showed differences across conditions. Specifically, SRs were better able to detect when the face was the same than the other groups and showed a more complex pattern in terms of their accuracy and response times. While a previous study found SRs were overall better than controls at identifying eye, mouth, and nose changes than controls on the part–whole task, a large number of SRs showed a bias towards nose changes [30]. We replicated this nose-superiority-effect and showed that SRs were generally more accurate than controls on the nose trials (see Figure 2C). However, after taking into account SRs’ slower response times for these trials (using balanced integration scores), no significant differences for the nose condition were observed. Indeed, SRs appear to have slower response times for all individual feature change trials (but not for whole face changes or repetitions), suggesting a more careful and time-consuming appraisal of these more difficult single feature change trial types than the other groups. The trend towards worse performance on the eye-change condition accompanied by superior accuracy for the nose condition in SRs suggests that SRs tend to fixate more on the nose area than controls (in line with the findings of a previous study; [31]). SRs’ numerically superior accuracy for the mouth trials is also consistent with this proposed difference in task strategy, though this apparent difference was a non-significant trend. The only condition in which SRs performed better in terms of both accuracy and response times was when identifying face repetitions. Further, examining the individual differences in accuracy and balanced integration scores, there was a larger variation in detecting individual feature changes in the SRs than in the controls and the DPs. This suggests that individual SRs may utilise different strategies on this task.

Post-hoc tests showed that the only condition that could distinguish between all three participant groups (DPs, controls, and SRs) was the face-repetition condition. Differences between controls and DPs for identifying face repetitions have previously been found by others (e.g., [25]). In this study, DPs incorrectly reported identity changes when faces were repeated (24%), while controls showed remarkably few such errors (5%). This has also been observed in another study which found the DPs were impaired in face-repetition trials but not face-change trials [44]. We suggest that DPs have difficulty with identifying when two faces are the same (i.e., “telling faces together”), even when these images are physically identical to one another (apart from a modest change in size). The finding that the face-repetition condition is the one condition in which SRs were overall reliably superior to the other groups (alongside the finding of higher accuracy for the nose-change condition than controls) also suggests that SRs have a disproportionate superiority in “telling faces together”. SRs are extremely good at identifying faces across large changes between comparison images [24,25,26,27], and therefore it is perhaps not surprising that they should find this condition easy—indeed, average accuracy for SRs on this condition was 97% and they had the fastest reaction times (534 ms). Taken together, it may be the case that the vast differences in face recognition ability observed between our three groups can most reliably be accounted for by the ability to tell that two images depict the same person rather than the ability to notice subtle differences between face images.

Interestingly, our data suggest that SRs’ superior accuracy in detecting certain feature changes may in part be strategic. SRs have superior accuracy for nose trials while also having slower response times for these single feature change trials. This pattern suggests a trade-off between speed and accuracy on these trials. Given that SRs also perform generally better on the same-face trials (both faster and more accurate), we suggest that this speed-accuracy trade-off is due to SRs’ specific awareness of these difficult trial types and is suggestive of increased diligence in these conditions. Interestingly, this increased diligence for nose and mouth changes appears to come at least in part at the expense of performance for the easier eye-change condition. If superior face-recognition ability is accompanied by strategic differences in the way in which face-processing tasks are completed, it opens the possibility that these strategies could be learned by others. Along with previous evidence for a “nose-superiority effect” in SRs [30], we suggest (along with others, e.g., [53]) that fixation at the centre of face images allows for more information to fall on the sensitive central retinal area which contains the highest visual acuity. Therefore, fixation around the nose may be an optimal strategy for completing face matching tasks [53,54] and might also allow for a more effective spread of visual attention across the whole face. Whether this optimal strategy can be learned via intervention, and whether those individuals with poor or normal face-recognition ability can benefit from this approach remains to be determined. Indeed, previous intervention studies have used the qualitative differences found in face perception between controls and individuals with DP in an attempt to improve face recognition, such as distances between features [55,56], and detecting subtle whole-face changes through the use of morphing techniques [57,58] (see [59,60] for reviews).

One limitation of this study is that it was not possible to measure response biases. It was not feasible to adequately employ signal-detection theory (i.e., d’prime) with these data, as, unlike other studies in which same/different decisions are made, “hits” can only occur in one condition in 20% of trials (“same face”) and “false alarms” can occur in the remaining four feature-change conditions. Therefore, it is not possible to clearly identify response biases in this study (i.e., would a response bias be deviation from 50/50 responding or 20/80 responding?). Further studies should examine this, and it would be interesting to compare any response biases between prosopagnosics and super-recognisers.

## 5. Conclusions

In conclusion, we found that although prosopagnosics were slower at identifying face feature changes as compared to controls, they showed a similar pattern of performance for the different feature changes. In contrast, super-recognisers showed differences in their patterns of response, with greater accuracy and faster response times for detecting nose changes than controls and were better able to detect when the face was the same. Finally, we found that the ability to identify when the same face was presented distinguished between all three groups. Our results provide further insight into the way that developmental prosopagnosics and super-recognisers identify changes between faces, with DPs being overall worse at the task, and SRs using a qualitatively different strategy.

## Figures and Tables

**Table 1:** Participant demographics.

		Gender						Age	
		Male		Female		Non-Binary			
	*n*	*n*	%	*n*	%	*n*	%	Mean	SE
Controls	33	12	(36)	19	(58)	2	(6)	42.8	(2.0)
DPs	33	4	(12)	29	(88)	0	(0)	43.2	(1.4)
SRs	33	12	(36)	20	(61)	1	(3)	41.8	(1.4)
ALL	99	28	(28)	68	(69)	3	(0.03)	42.6	(0.9)

**Table 2:** Main effect of Condition for super-recognisers (SRs) and controls. Tukey’s post-hoc *t*-test; df = 128. Bold = significant.

		Controls			SRs		
		MD	*t*	p_tukey_	MD	*t*	p_tukey_
diff	same	0.02	0.35	1.00	−0.01	−0.20	1.00
	eyes	**0.12**	**2.77**	**0.05**	**0.25**	**4.50**	**<0.001**
	nose	**0.71**	**16.29**	**<0.001**	**0.53**	**9.70**	**<0.001**
	mouth	**0.60**	**13.92**	**<0.001**	**0.51**	**9.30**	**<0.001**
same	eyes	0.10	2.42	0.12	**0.26**	**4.70**	**<0.001**
	nose	**0.69**	**15.94**	**<0.001**	**0.55**	**9.89**	**<0.001**
	mouth	**0.59**	**13.57**	**<0.001**	**0.52**	**9.49**	**<0.001**
eyes	nose	**0.59**	**13.52**	**<0.001**	**0.29**	**5.20**	**<0.001**
	mouth	**0.48**	**11.15**	**<0.001**	**0.26**	**4.80**	**<0.001**
nose	mouth	−0.10	−2.38	0.13	−0.02	−0.40	1.00

**Table 3:** Group differences between super-recognisers and controls for each condition. Note there was a significant interaction between Group and Condition. Independent samples *t*-tests; df = 64. Bold = significant; italics = trend.

	*t*	*p*
diff	*1.75*	*0.09*
same	**3.31**	**0.00**
eyes	*−1.79*	*0.08*
nose	**2.99**	**0.00**
mouth	1.43	0.16

**Table 4:** Main effect of Condition for controls and prosopagnosics. Tukey’s post-hoc *t*-test; df = 256. Bold = significant.

		MD	*t*	p_tukey_
diff	same	0.01	0.42	0.99
	eyes	**0.13**	**4.22**	**<0.001**
	nose	**0.69**	**22.89**	**<0.001**
	mouth	**0.59**	**19.49**	**<0.001**
same	eyes	**0.11**	**3.80**	**0.00**
	nose	**0.68**	**22.47**	**<0.001**
	mouth	**0.57**	**19.07**	**<0.001**
eyes	nose	**0.56**	**18.67**	**<0.001**
	mouth	**0.46**	**15.27**	**<0.001**
nose	mouth	**−0.10**	**−3.39**	**0.01**

**Table 5:** Main effect of Condition for super-recognisers (SRs) and controls. Tukey’s post-hoc *t*-test; df = 128. Bold = significant.

		Controls			SRs		
		MD	*t*	*p*	MD	*t*	*p*
diff	same	−0.22	−1.32	0.68	−0.28	−1.20	0.75
	eyes	0.46	**2.81**	**0.04**	**1.47**	**6.33**	**<0.001**
	nose	1.96	**11.95**	**<0.001**	**2.21**	**9.48**	**<0.001**
	mouth	1.89	**11.57**	**<0.001**	**2.45**	**10.52**	**<0.001**
same	eyes	0.68	**4.14**	**<0.001**	**1.75**	**7.53**	**<0.001**
	nose	2.17	**13.28**	**<0.001**	**2.49**	**10.68**	**<0.001**
	mouth	2.11	**12.89**	**<0.001**	**2.73**	**11.72**	**<0.001**
eyes	nose	1.50	**9.14**	**<0.001**	**0.73**	**3.15**	**0.02**
	mouth	1.43	**8.75**	**<0.001**	**0.98**	**4.19**	**<0.001**
nose	mouth	−0.06	−0.39	1.00	0.24	1.04	0.84

**Table 6:** Group differences between super-recognisers and controls for each condition. Note there was a significant interaction between Group and Condition. Independent samples *t*-tests; df = 64. Bold = significant; italics = trend.

	*t*	*p*
diff	1.47	0.15
same	**2.41**	**0.02**
eyes	*−1.93*	*0.06*
nose	0.25	0.80
mouth	−0.75	0.46

**Table 7:** Group differences between prosopagnosics and controls for each condition. Note there was not a significant interaction between Group and Condition. Independent samples *t*-tests; df = 64. Bold = significant; italics = trend.

	*t*	*p*
diff	*1.82*	*0.07*
same	**2.75**	**0.01**
eyes	**2.31**	**0.02**
nose	**3.43**	**<0.001**
mouth	**2.78**	**0.01**

**Table 8:** Main effect of Condition for controls and prosopagnosics. Tukey’s post-hoc *t*-test; df = 256. Bold = significant.

		MD	*t*	*p*
diff	same	−0.13	−0.96	0.87
	eyes	0.57	**4.40**	**<0.001**
	nose	2.19	**16.80**	**<0.001**
	mouth	2.00	**15.39**	**<0.001**
same	eyes	0.70	**5.36**	**<0.001**
	nose	2.31	**17.76**	**<0.001**
	mouth	2.13	**16.35**	**<0.001**
eyes	nose	1.61	**12.40**	**<0.001**
	mouth	1.43	**10.99**	**<0.001**
nose	mouth	−0.18	−1.41	0.62

## Data Availability

The original contributions presented in the study are included in the article; further inquiries can be directed to the corresponding author.

## Supplementary Materials

The following supporting information can be downloaded at: https://www.mdpi.com/article/10.3390/brainsci14060561/s1, The following supporting information are seen in the supplementary section at the end of the manuscript. Main effect of Condition for super-recognisers (SRs) and controls. Tukey’s post-hoc *t*-test; df = 128. Bold = significant; italics = trend. Table S2. Group differences between prosopagnosics and controls for each condition. Note there was not a significant interaction between Group and Condition. Independent samples *t*-tests; df = 64. Bold = significant; italics = trend. Table S3. Main effect of Condition for controls and prosopagnosics. Tukey’s post-hoc *t*-test; df = 256. Bold = significant; italics = trend.
