# Supplementary material for: Face Feature Change Detection Ability in Developmental Prosopagnosia and Super-Recognisers
Source: Brain Sci. 2024 May 30;14(6):561. doi: 10.3390/brainsci14060561 (PMC11201608; doi:10.3390/brainsci14060561)
Supplement: Supplementary file 1 [file brainsci-14-00561-s001.zip › brainsci-3025353-supplementary.pdf]

SUPPLEMENTARY MATERIAL – REACTION TIME ANALYSIS

Reaction times

A 3 (group; between subjects) x 5 (condition; within subjects) ANOVA revealed a main effect of Group ($F(2,96) = 4.94$, $P = 0.009$), Condition ($F(4,384) = 22.33$, $P < 0.001$), and an Interaction ($F(8,384) = 4.24$, $P < 0.001$). As we were interested in how the two groups (DPs and SRs) differed in their response to controls, we examined these differences by conducting 2 x 5 ANOVAs for super-recognisers versus controls, and another for prosopagnosics versus controls.

Super-recognisers compared to controls

For SRs versus controls, there was an effect of Condition ($F(4,256) = 21.85$, $P < 0.001$) and an Interaction ($F(4,256) = 8.51$, $P < 0.001$), but no effect of Group ($F(1,64) = 0.74$, $P = 0.39$). Due to the effect of Condition and Interaction, we then conducted a 1 x 5 ANOVA for controls and SRs separately. This showed an effect of condition for both controls ($F(4,128) = 5.40$, $P < 0.001$) and SRs ($F(4,128) = 18.90$, $P < 0.001$). The effect of Condition was explored using Tukey's post-hoc t-tests (**Supplementary Table S1**) which showed different patterns of results for controls and SRs. Specifically, SRs showed no significant differences in reaction times between the eyes, nose, and mouth, or between whole face changes or the same face. Meanwhile, controls only showed differences in reaction times between the same and eye changes, and the same and mouth changes.

Supplementary Table S1. Main effect of Condition for super-recognisers (SRs) and controls. Tukey's post-hoc t-test; df = 128. Bold = significant; italics = trend.

		Controls			SRs		
		MD	t	p_{tukey}	MD	t	p_{tukey}
diff	same	80.00	*2.65*	*0.07*	73.30	1.50	0.57
	eyes	-33.30	-1.10	0.80	-222.90	**-4.55**	**< .001**
	nose	36.08	1.20	0.75	-193.60	**-3.95**	**0.00**
	mouth	-37.64	-1.25	0.72	-271.10	**-5.54**	**< .001**
same	eyes	-113.30	**-3.76**	**0.00**	-296.20	**-6.05**	**< .001**
	nose	-43.92	-1.46	0.59	-266.90	**-5.45**	**< .001**
	mouth	-117.64	**-3.90**	**0.00**	-344.40	**-7.03**	**< .001**
eyes	nose	69.37	2.30	0.15	29.30	0.60	0.98
	mouth	-4.34	-0.14	1.00	-48.20	-0.99	0.86
nose	mouth	-73.71	-2.45	0.11	-77.50	-1.58	0.51

Developmental prosopagnosics compared to controls

For DPs versus controls, there was an effect of Group ($F(1,64) = 11.10$, $P = 0.001$), an effect of Condition ($F(4,256) = 6.56$, $P < 0.001$), but only a trend for an Interaction ($F(4,256) = 2.19$, $P = 0.07$). The effect of Group with no Interaction was caused by overall faster reaction times in controls compared to DPs; however, we have included these comparisons for reference in **Supplementary Table S2**.

Supplementary Table S2. Group differences between prosopagnosics and controls for each condition. Note there was *not* a significant interaction between Group and Condition. Independent-samples t-tests; df = 64. Bold = significant; italics = trend.

	t	p
diff	*-1.72*	*0.09*
same	**-2.93**	**0.005**
eyes	**-2.51**	**0.015**
nose	**-3.57**	**< .001**
mouth	**-2.85**	**0.006**

The effect of Condition was explored using Tukey's post-hoc t-tests collapsed across groups (**Supplementary Table S3**) which showed significantly faster reaction times for same face than for eye changes, nose changes, and mouth changes (P's < 0.05), as well as faster reaction times for different face than mouth changes (P < 0.05). There were no other differences in reaction times.

Supplementary Table S3. Main effect of Condition for controls and prosopagnosics. Tukey's post-hoc t-test; df = 256. Bold = significant; italics = trend.

		MD	t	p
diff	same	49.70	1.68	0.45
	eyes	-60.70	-2.05	0.24
	nose	-48.20	-1.63	0.48
	mouth	-84.40	**-2.85**	**0.04**
same	eyes	-110.50	**-3.73**	**0.00**
	nose	-97.90	**-3.31**	**0.01**
	mouth	-134.10	**-4.53**	**< .001**
eyes	nose	12.60	0.43	0.99
	mouth	-23.60	-0.80	0.93
nose	mouth	-36.20	-1.22	0.74

Results summary

In sum, when comparing controls and SRs, SRs showed no significant differences in reaction times between the eyes, nose, and mouth, or between whole face changes or the same face, while controls only showed differences in reaction times between the same face and eye changes, and the same face and mouth changes. When comparing controls to DPs, controls had overall faster reaction times than DPs. Both controls and DPs had faster reaction times for same face than for eye changes, nose changes, and mouth changes, as well as faster reaction times for different face than mouth changes.
